# A Successful Method of Attaining Traction in Above-Knee Amputation Patients With Intertrochanteric Hip Fractures

**DOI:** 10.7759/cureus.64157

**Published:** 2024-07-09

**Authors:** David P Essex, Sami Alaraj, Vinod Panchbhavi

**Affiliations:** 1 John Sealy School of Medicine, University of Texas Medical Branch, Galveston, USA; 2 Department of Orthopedic Surgery and Rehabilitation, University of Texas Medical Branch, Galveston, USA

**Keywords:** traction, lower extremity, intertrochanteric fractures, fracture fixation, amputation

## Abstract

Intertrochanteric fractures are a common occurrence in the general population; however, in patients with above-knee amputations, they are relatively rare. In this patient population, positioning on a fracture table presents a particularly difficult problem prior to the fixation of an intertrochanteric fracture. Here, we describe a 57-year-old man with extensive vasculopathy and reduced bone density who presents with an intertrochanteric fracture after a fall from standing. Adequate traction of the amputated leg was achieved via the modification of a standard fracture table and the utilization of a Bohler traction bow. Fixation of the intertrochanteric fracture was successful, and the patient suffered no postoperative complications.

## Introduction

Hip fractures are a common cause of hospitalizations, with around 300,000 admissions a year in the US. Of these, intertrochanteric fractures (ITF) account for almost 50% of all hip fractures [1,2]. The mechanism of injury is commonly due to a fall from standing and occurs most often in the elderly population, although younger patients who smoke, have chronic diseases, abuse alcohol, and have low education are at an increased risk [3].

Historically, ITF has been treated operatively with a sliding hip screw fixation or intramedullary nailing (IMN), though the latter has become more prevalent in recent years [4-6]. Prior to IMN placement, closed reduction of the fracture is achieved by applying axial traction of the lower extremity along with internal or external rotation and adduction or abduction of the affected limb [7]. Further, imaging of the contralateral limb can be used as a guide for appropriate alignment of the injured femur [8].

Patients with an amputated limb, however, present a particular challenge to the traditional use of a fracture table and necessitate a unique approach. We present a method of utilizing a Bohler traction bow to facilitate traction with rotational control in a rare case of a patient with a left-sided ITF with a prior left-sided above-knee amputation (AKA).

This article was presented as a poster at the Inaugural Orthopedic Research Forum hosted by the University of Texas Medical Branch on May 17, 2024.

## Case presentation

**Figure 1:**
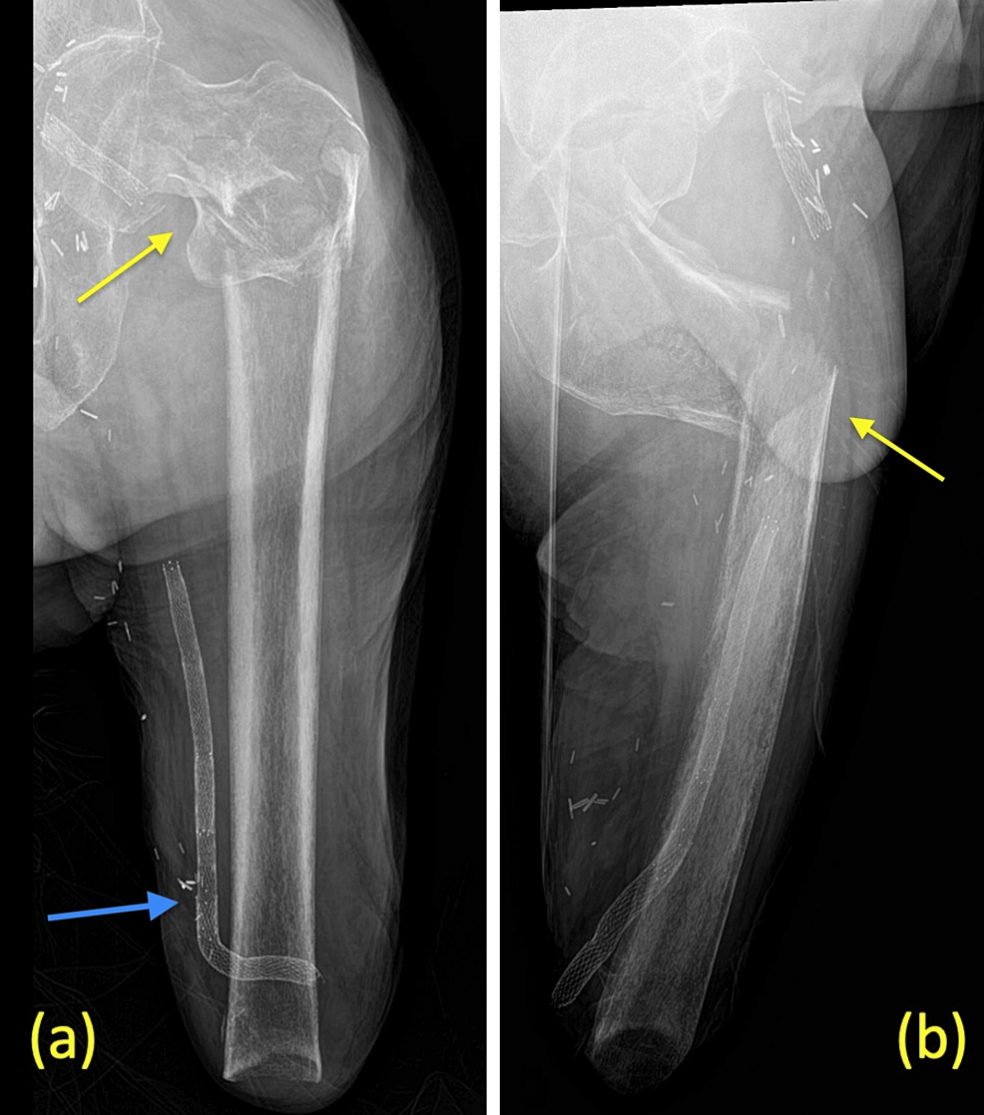
Radiograph imaging a: anteroposterior view of the left femur showing a comminuted intertrochanteric fracture with subtrochanteric extension (yellow arrow), amputated femur, and vascular graft (blue arrow); b: lateral view of the left femur showing anterior displacement of the fracture.

A 57-year-old male presented to the emergency department with severe left hip pain following a fall from standing. His past medical history is significant for a left-sided below-knee amputation (BKA) that was subsequently revised to an AKA, coronary artery disease, a history of myocardial infarction, a 30-pack-year smoking history, and extensive vasculopathy with the presence of a vascular graft near the amputation stump. The patient is not active in everyday life and does not use a prosthesis. He ambulates with a walker at home when needed. Radiographic imaging revealed a displaced, unstable ITF with the subtrochanteric extension of the left hip (Figures 1a, 1b). Treatment options were discussed with the patient given his limited mobility, and a physician-patient shared decision to proceed with surgery due to severe pain was elected. Due to his cardiovascular history, the patient was seen and cleared by cardiology preoperatively.

**Figure 2:**
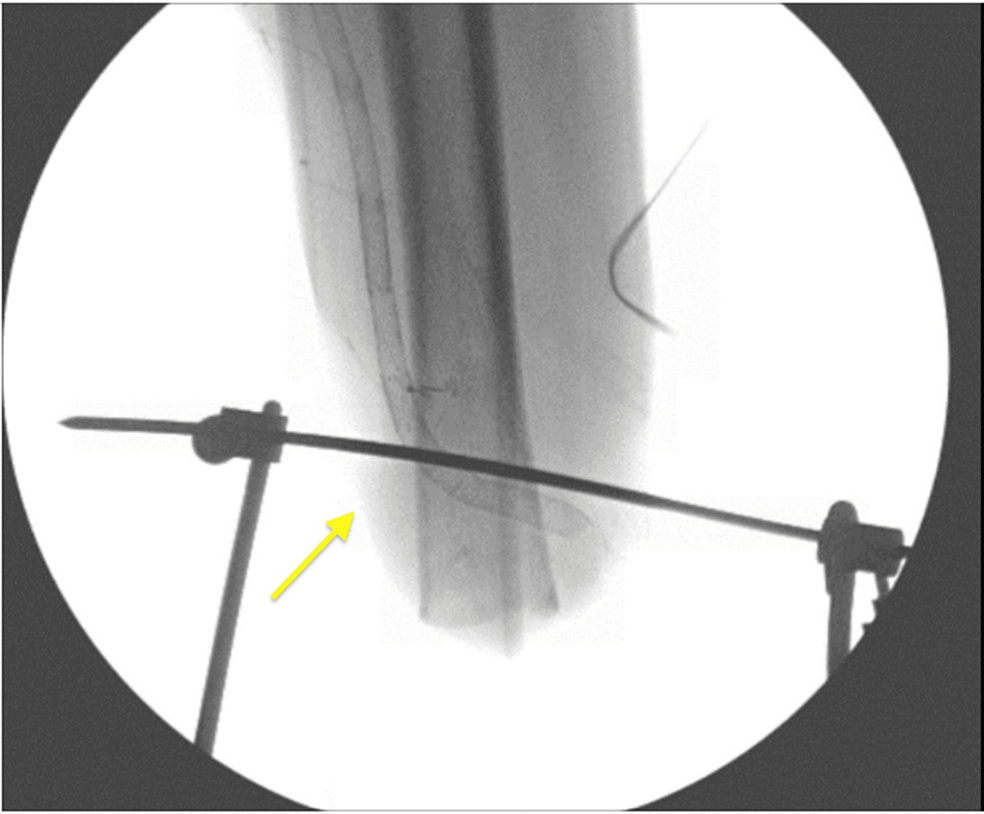
Fluoroscopic image showing position of traction pin and proximity to stump

**Figure 3:**
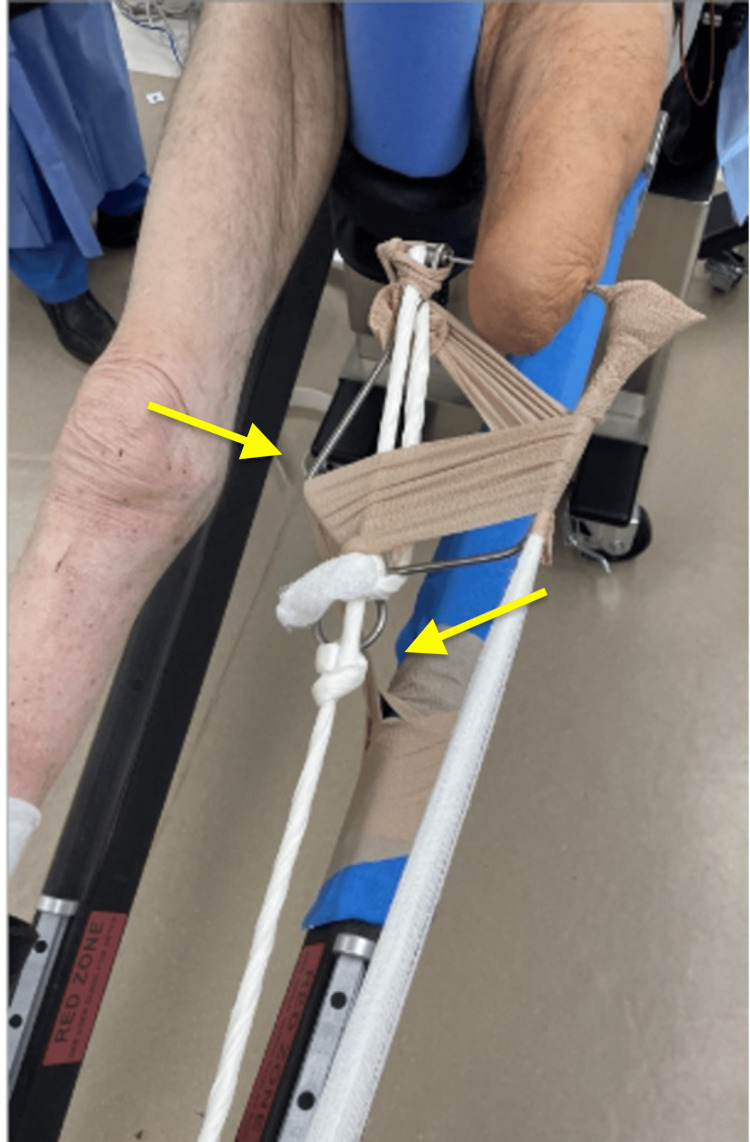
Picture showing traction pin and Bohler traction bow with gauze fortified with silk tape and self-adhering bandage used to control rotation attached to the traction arm

The patient was then transferred to the operating room, where he underwent general anesthesia. Once intubated, he was placed on a fracture table for positioning. C-arm fluoroscopy was available in the room. The operative leg was short due to AKA, and it presented a challenge as it would not fit in the available traction attachments. The decision was made to use skeletal traction at the distal femur and attach it to the fracture table traction arm. A 2-mm Steinmann pin was drilled percutaneously from medial to lateral at the distal femur under fluoroscopic guidance, and imaging was obtained to confirm the position (Figure 2). Next, a Bohler traction bow was applied over the pin, and gauze overwrapped with silk tape was used to tie the bow to the traction arm at the end of the table. The traction mechanism was successful, and an adequate reduction was obtained and confirmed by fluoroscopy. A self-adhering cohesive bandage was used at the traction bow to help control rotation (Figure 3).

**Figure 4:**
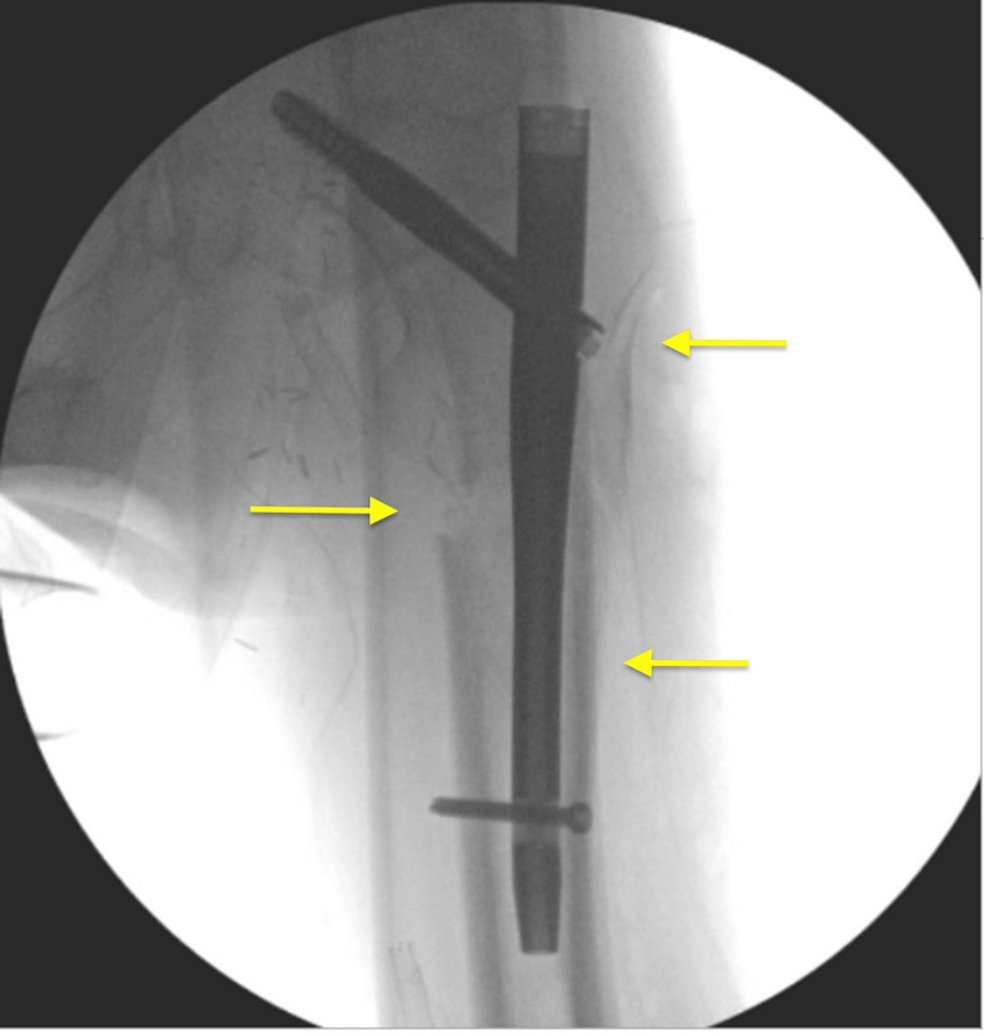
The final fluoroscopic anteroposterior image shows improved alignment and the appropriate position of the implant

Next, the patient was draped appropriately, and we proceeded with the IMN portion of the procedure. A small lateral incision was made 5 cm proximal to the greater trochanter (GT), and dissection was carried through the iliotibial band down to the tip of the GT. Next, a 3.2 mm Kirschner wire was used to obtain a trochanteric entry point. The opening reamer was then used, followed by the insertion of the guidewire. The positioning of the wire was adequate, as confirmed by AP and lateral views. The length of the wire was then measured. The decision was made to proceed with a size 11 mm x 180 mm, 125-degree short femur nail after measuring the canal diameter preoperatively. The canal was reamed with a 12 mm reamer, and the nail was then inserted. Imaging confirmed adequate placement, and the guidewire was removed. Next, the cephalomedullary screw was inserted, traction was released, and the distal interlocking screw was inserted. Final imaging was obtained, showing acceptable reduction and placement of the femoral nail. The wound was then irrigated, and hemostasis was obtained. Next, the incision was closed in a layered fashion using 0 and 2-0 Vicryl and staples for skin. A sterile dressing was then applied. Next, the Steinmann pin was removed from the distal femur, and a sterile dressing was then applied. Postoperative radiographs showed stable reduction and fixation of the fracture (Figure 4). On postoperative day one, the patient was seen and examined and reported improvement in his hip pain. The patient recovered and had a favorable outcome, with no known complications following the surgery.

Attaining regular follow-up with the patient was difficult due to distance and his inability to travel to the clinic. He was contacted about follow-up, but he communicated that he preferred to follow up closer to home. At last communication 30 days post-op, his pain was tolerated, and he had returned to his normal activity level. The patient was then lost to follow-up and has not responded to recent communication attempts. All information in this case report has been de-identified to protect privacy.

## Discussion

While the use of fracture tables is ideal for most patients, those with a lower limb amputation warrant special consideration for positioning and traction. Given the relative infrequency of hip fractures in this patient population, guidance on managing such cases is lacking in the current literature.

Previously published case reports of hip fracture patients with BKA have suggested inverting the traction table boot or utilizing a proximal tibial pin to attain traction [9,10]. These techniques depend on the anatomy and strength of an intact knee joint, which presents an additional challenge in achieving adequate traction in patients with AKA.

There are few examples of cases of ITF in patients with AKA. Aqil et al. reported using a radiolucent leg support to aid in the fixation of a minimally displaced ITF [11]. Davarinos et al. published a report on their experience using skin traction in a nondisplaced ITF [12]. Similarly, Lee et al. also described utilizing skin traction to reduce ITF [13]. These methods were all successful in their implementation. However, it is important to note that they all treated nondisplaced or minimally displaced fractures; the techniques utilized in these cases may not be able to withstand the force of traction needed in more severe fractures. Further, as noted by Davarinos et al., the skin traction technique was limited in terms of rotational capability and presented a risk for tearing of the skin in certain patients.

Takeba et al. described a successful method in which they treated a displaced ITF in a patient with AKA by inserting a 2.3 mm Kirschner wire through the distal femur and attaching a horseshoe and traction rope to the fracture table [14]. This allowed them to achieve an adequate reduction of the fracture for subsequent IMN. This was done in the setting of a high-impact motor vehicle collision that caused a crush injury necessitating a traumatic amputation. It is unknown whether their method would be as successful in patients with low bone density or with multiple comorbidities. Further, the traction rope attachment they used may not be able to provide adequate control over rotation.

Similar to the above cases, the technique we presented was successful in providing a closed reduction of our patient's ITF. However, our case is unique in that our patient had multiple comorbidities affecting his bone health that provided additional challenges for positioning, including his prior amputation, smoking history, and cardiovascular health. Unilateral AKA is associated with reduced bone density in the affected leg when compared to the normal side, particularly around the femoral neck [15]. Additionally, the patient had a 30-pack-year smoking history and extensive cardiovascular disease, including hypertension, hyperlipidemia, coronary artery disease with a history of stent placement, and severe peripheral arterial disease with the presence of a vascular graft. Both smoking and cardiovascular disease have been shown to affect bone density negatively [16,17]. Because of this, we opted to attach the traction bow to a smaller 2-mm non-threaded Steinmann pin to minimize the risk of pull out.

Our technique has multiple benefits that allow for successful treatment. The use of a distal femur pin and Bohler traction bow allowed for robust traction, and using the self-adhering cohesive bandage attached to the spar helped with rotational control. None of the other techniques discussed above described an adequate method of controlling rotation, which is key to achieving proper reduction. Furthermore, there is less risk of skin damage when compared to skin traction. Finally, this technique has the benefit of requiring only minimal resources, with which most operating rooms are equipped, increasing its usability.

Although our patient experienced no known complications, there are a few potential points of concern to address.

First, and not unique to our technique, is the risk of infection at the site of insertion of the distal femoral pins. Pin site infection is a common complication with variable incidence and no standardized prophylaxis or treatment course [18]. Because of this, we suggest maintaining the sterile field at all times and promptly removing the pins when no longer needed.

Another potential complication is the possibility of the distal femur pin to pull out during traction, which has been noted in other cases [12]. The risk is particularly relevant for patients with AKA, as discussed above. For this reason, an assessment of the risks and benefits of inserting a pin in the distal femur should be considered on an individual basis before attempting this technique.

Finally, the insertion of the distal femur pin and subsequent tractional force applied to the AKA stump has the potential to damage the surrounding soft tissues, leading to neuropathic pain or sensitivity to the area.

## Conclusions

Proper positioning is paramount to obtaining an adequate reduction of intertrochanteric fractures. However, in certain patient populations, such as those with above-knee amputations, positing is complicated by the lack of adaptability of a traditional fracture table. Our case demonstrates an effective method of modifying a fracture table to facilitate internal reduction of an intertrochanteric fracture with subtrochanteric extension in a patient with an above-knee amputation, extensive vasculopathy, and poor bone health. Further studies testing the safety and efficacy of our technique are needed to determine its validity in this general patient population.
